# Effectiveness of cognitive behavioural therapy and social skills training in management of conduct disorder

**DOI:** 10.4102/sajpsychiatry.v28i0.1737

**Published:** 2022-07-22

**Authors:** Daniel O. Kumuyi, Ebenezer O. Akinnawo, Bede C. Akpunne, Aderonke A. Akintola, Deborah F. Onisile, Onyeka O. Aniemeka

**Affiliations:** 1 Department of Behavioural Studies, Faculty of Social Sciences, Redeemer’s University, Ede, Osun State, Nigeria; 2 Department of Nursing Science, Faculty of Basic Medical Sciences, Redeemer’s University, Ede Osun State, Nigeria; 3 Department of Psychology, Federal Neuropsychiatric Hospital Yaba, Lagos, Nigeria

**Keywords:** cognitive behavioural therapy, social skill training, conduct disorder, adolescents, Nigeria

## Abstract

**Background:**

Conduct Disorder (CD) is a repetitive disruptive behaviour that violates the rights of others, manifests in rules violation, aggression, hostility, and deceitfulness and has assumed prominence in its association with juvenile offending and criminality in adulthood. Despite this knowledge, little research attention is given to ascertaining effective psychobehavioural interventions to manage this problem, especially amongst Nigerian adolescents.

**Aim:**

This study examined the efficacy of two psychobehavioural strategies to manage CD amongst in-school adolescents in Ibadan, Nigeria.

**Setting:**

Ibadan, Southwestern Nigeria.

**Method:**

A randomised controlled trial (RCT) of adolescents with CD was performed. Sixteen participants (aged 12–17 years) who reported high CD from an assessment of 1006 in-school adolescents of selected secondary schools in Ibadan were randomly grouped to receive either cognitive behavioural therapy (CBT), social skills training (SST) or combined CBT and SST. The Frequency of Delinquent Behaviour Scaling Instrument (FDBSI) was used for assessments.

**Results:**

Significant reduction in CD was observed among participants exposed to CBT (*t*[6] = 8.510), *p* < 0.05) at 8 weeks, SST (*t*[6] = 12.728), *p* < 0.05) at 8 weeks, and combined CBT and SST (*t*[8] = 12.728, *p* < 0.05) at the 6 week mark of interventions respectively.

**Conclusion:**

From the study, CBT and SST are effective in managing CD. However, the combined psychobehavioural intervention of CBT and SST was more effective in managing CD, based on a faster therapeutic effect than the independent psychobehavioural intervention of CBT and SST.

## Introduction

Conduct disorder (CD) is defined as a repetitive and persistent pattern of behaviour that violates the rights of others or that violates major age-appropriate societal norms or rules.^1^ It is typically diagnosed in adolescents under the age of 18 and is often a precursor to antisocial personality disorder, according to the Diagnostic Statistical Manual of Mental Disorders (DSM-5). Some symptoms of CD include bullying, threatening, intimidating, initiating fights, using a weapon to cause harm, cruelty toward people and/or animals, stealing whilst confronting a victim, forcing into sexual activity, fire-setting, destroying property, breaking into property, lying to obtain goods or avoid obligations and shoplifting.^1,2^ Other rule violations include staying out late despite parenting expectations, running away overnight without returning for a lengthy period and truancy onset before age 13.^1^ If a child shows symptoms prior to age 10, it is classified as childhood-onset type. If not, it is classified as adolescent-onset type.

Studies have suggested that CD is most rampant amongst children and adolescents.^3^ Adolescence is a time in human life when many changes occur and is characterised by some sporadic physical growth and physiological changes. It is also a period of cognitive, social and contextual transitions.^1^ The adolescent period is a modern concept in society, leading to prolonged childhood through lengthy adolescence. It is a time when the individual attends secondary school or learns a trade.^4^ The period is a time of strain and stress fraught with many problems. Thus, it is characterised by instability and susceptibility to the development of psychological distress, which may lead to engaging in delinquency to reduce or escape from the strain they are experiencing.^5^ Previous research also established that the ‘stress and storm’ that adolescents go through make them experience mood disruptions, risky behaviour and conflicts with parents. These three characteristics affect their emotional, social and physical interactions with others.^6^

Various behavioural modification techniques like cognitive-behavioural therapy (CBT) and social skills training (SST), amongst others, have been used to treat rebelliousness, disorderliness and other disruptive behaviours.^7,8^ However, the efficacy of most of these techniques on CD, especially amongst in-school adolescents, is yet to be empirically established in Nigeria.

Cognitive-behavioural therapy helps children and adolescents to learn better ways to manage their anger and solve social problems by increasing emotion-regulation and problem-solving abilities.^9,10^ In particular, children and adolescents learn to identify their level of anger and use coping self-statements, distraction techniques and brief deep-breathing relaxation methods to handle arousal associated with their anger. They also learn and improve skills to adequately interpret social problems, generate possible solutions and decide which solution will be implemented. In contrast, SST uses direct instruction to teach specific skills through modelling, role-playing, corrective feedback and practice.^11^ In addition to teaching specific skills,^12^ it also indicates the need to remove competing behaviours and facilitate generalisation and maintenance.^13^ Furthermore, SST programmes commonly emphasise the increase of acquisition, performance, generalisation and maintenance of prosocial behaviours and the decrease of antisocial behaviours.

Social skills training aims to decrease disruptive behaviour and increase on-task behaviour and social problem-solving skills.^14^ Cognitive-behavioural therapy emphasises specific cognitive techniques designed to produce changes in thinking that result in changes in behaviour.^15^ The future of CBT may involve its integration with other types of approaches. Integrating CBT with strengths-based approaches may similarly yield improved outcomes.^16^ This type of integration may be significant for achieving enhanced outcomes amongst adolescents with conduct problems. For instance, SST was included in a meta-analysis on treatment effectiveness for juvenile offenders aged 12–21; it was categorised as a skill-building programme when used alongside CBT interventions.^17^ This skill-building programme was found to result in 12% less recidivism than a control group with a 50% recidivism rate, even when controlling for study design and demographic characteristics. In view of the foregoing, this study sought to examine the efficacy of CBT and SST, both separately and combined, in the management of in-school adolescents with CD in the metropolis of Ibadan, southwestern Nigeria. Four hypotheses were generated to guide the study.

### Hypotheses

- Participants who received CBT intervention will significantly score lower on the measure of CD than those in the control group.
- Participants who received SST intervention will score substantially lower on the measure of CD than those in the control group.
- Participants who received combined cognitive behaviour therapy and SST interventions will significantly score lower in the measure of CD than those in the other treatment groups and the control group.
- Combined CBT and SST will be more efficacious than each treatment received alone.

## Methods and materials

### Participants

The study population are in-school adolescents of secondary schools in Ibadan, Oyo State, Southwestern Nigeria. A total of 1006 adolescents selected from three junior secondary schools and three senior secondary schools participated in the study.^18^

A quasi-experimental design was employed in this study. In line with^19^ the recommendation that 50% of the target population must be used for a study to be representative, a multi-stage sampling technique was employed in this study. The ballot technique was used to randomly select three out of the five Local Government Areas (LGAs) in the Ibadan metropolis. Schools in the metropolis already existed in stratified form in each local government as public and private. Next, a convenient sampling technique was employed in selecting one public and private school from each of the three LGAs. Finally, a systematic sampling technique was employed in selecting the adolescents who were willing to participate in the survey study. The samples’ breakdown of participants who exhibited high levels of CD from the six schools in the second phase (main study) of the study were 15 students from Adesina College, 16 students from Anglican Grammar School and 20 students from Bishop Philips Academy. Also, 14 students were selected from IMG Grammar School, 15 from Oritamefa Baptist Junior and Secondary School and 17 students from St. Patrick Grammar School. However, the only school with the highest number of 20 was used for the intervention.

The data from the fieldwork were subjected to SPSS analysis. The schools’ names for the study were written on the different batteries of filled (answered) questionnaires and coded. Hence, from the analysis, the school with the highest number of students who scored the highest on CD was used for the intervention. Twenty of the students from this selected school met the criteria for this study. The previous research result was briefly discussed with them, and parental consent forms were requested from the willing participants. Out of 20 participants, only 16 parents agreed to participate by signing the consent form, and the students themselves filled in the assent form.

### Measures

The Frequency of Delinquent Behavior Scaling Instrument (FDBSI) was developed by the Center for Disease Control and Prevention in the United States of America (USA).^20^ It is a 25-item instrument with six subscale measures, namely, vandalism, theft, physical aggression, truancy, disruptiveness and status offence. Items 1–3 measure vandalism, items 4–10 measure theft, items 11–15 measure physical aggression, items 16–19 measure truancy, items 20–21 measure disruptiveness and items 22–25 measure status offence. The instrument has five response categories of ‘never’ = 0, ‘1–2 times’ = 1, ‘3–6 times’ = 2, ‘7–9 times’ = 3 and ‘more than 10 times’ = 4. Examples of items in the scale are as follows: ‘have you ever taken something from a store without paying for it?’ Composite scores for all the subscales were obtained. Norm score values were derived for both male and female participants in this study (≥28.4) (≥18.8), respectively. By implication, any individual score equal to or greater than the norm is considered as high CD. Such individual will require a psychological intervention. A Cronbach’s alpha value of 0.75 was derived from a pilot conducted prior to the study.^21^

### Procedure

The therapists involved in the study were licenced clinicians who had undergone supervised internships in psychotherapy using treatment manuals. They were of the speciality of clinical psychology.

The intervention stage was divided into five phases: the pretest, then weeks 2, 4, 6 and 8, the last of which served as the posttest. At this stage, the population of the study was 16 in-school adolescents with a severe level of CD identified from the cross-sectional assessment phase of the study.

The 16 participants were randomly assigned into four groups (control group and three intervention groups) using simple random sampling (ballot technique). There were four in each group: the control group, the CBT group, the SST group and the CBT & SST group. The intervention was for 8 weeks, having one session a week and eight sessions in all.

### Research setting

The study was carried out in a classroom of the school with the highest reported CDs, both in degree and number. This was based on our initial study of six selected secondary schools in Ibadan.

### Inclusion and exclusion criteria

For a participant to be included in the study, they were required to be between 10 and 18 years. Also, their parents must have signed the consent form, with the student filling in the assent form, and they must have participated in the first survey study. The student was required to meet the severe level of CD category (based on our prior assessment). Those without comorbid psychiatric conditions and those who had not received any mental health treatment were included.

Those excluded from the study were students who were below 10 years old or above 18 years of age. Also, those without a consent form from parents or who presented with major physical or intellectual disabilities were excluded from this study.

### Baseline screening

All consenting participants in this research were screened at their first appointment by the researchers. The screening involved the clinical assessment of all participants, ascertaining the level of CD and collecting the sociodemographic and baseline data of all consenting participants. To establish present levels of CD, the screening and assessment tools were re-administered to the 16 selected participants before the commencement of the study. This was taken as the baseline for comparison.

### The experimental group

A total of three junior secondary school students and nine senior secondary school students between the ages of 12–18, comprising eight boys and four girls, were categorised under the experimental group. This treatment group underwent 8 weeks of treatment sessions at the rate of 1 h per week, conducted by the researchers. This group participated in either CBT, SST or combined CBT and SST psychotherapy sessions for 8 weeks. The measurement of CD was taken at the baseline, week 2, mid-test (4 weeks), week 6 and posttest (8th week).

### The control group

This group did not participate in any psychotherapy. Two junior secondary school students and two senior secondary school students between the ages of 13–17, comprising three boys and a girl, were placed in the control group. They were students living with both parents and shared parameters with the treatment group. This control group had no placebo; they were just a wait-list group that came together during the 8 weeks intervention sessions of the treatment groups. The measurement of CD was taken at baseline, week 2, at mid-test (4 weeks), week 6 and posttest (8th week) for this group as well.

### Interventions modules

The three intervention modules designed for the experimental group are:

### Cognitive-behavioural therapy

1. Basic terms and concepts in cognitive restructuring and pretest administration
2. Psychoeducation
3. Identification of participants’ problems and information on CD
4. Overcoming CD through the principle of cognitive restructuring
5. Application of the problem-solving approach
6. Emphasis on the benefits of cognitive restructuring therapy for adequate restoration of expected behaviour outcome
7. General evaluation of the cognitive restructuring skill training
8. Wrap-up and posttest administration.

### Social skills training

1. Introduction of basic terms and concepts of SST and administration of pretest
2. Choices – problem solving
3. Tuning in – self-monitoring and emotions
4. Not losing it – regulating emotions, self-talk and coping
5. Speaking out – types of communication
6. Concretisation of the benefits of SST for adequate restoration of expected behaviour outcomes
7. General evaluation of the SST
8. Wrap-up and posttest administration.

### Cognitive-behavioural therapy + social skills training

1. Basic terms and concepts in cognitive restructuring and SST and pretest administration
2. Identification of participants’ problem and information on CD
3. Tuning in – self-monitoring and emotions
4. Overcoming CD through the principle of cognitive restructuring
5. Not losing it – regulating emotions, self-talk and coping
6. Emphasis on the benefits of cognitive restructuring therapy and SST for adequate restoration of expected behaviour outcomes
7. General evaluation of the cognitive restructuring and socials skill training
8. Wrap-up and posttest administration.

### Ethical considerations

The purpose of the research as well as the procedures were scrutinised and approved by the ethical research committee of Ministry of Education, Science and Technology, Schools Department, Oyo State Nigeria (reference number: EDU/188/VOL11T3/59). The researchers have undertaken psychotherapeutic supervision training at two reputable psychiatric hospitals in Nigeria. The research was carried out in conformity with the 1964 Declaration of Helsinki. Regarding international standards, participants were duly educated on the purpose and activities involved in the study. Confidentiality was assured and enrolment of participants was strictly voluntary. Moreover, parental consent forms and adolescent assent forms were signed and obtained before the commencement of this study.

## Analytical statistics and results

The finding summarised in Table 1 showed that at pretest level there was no significant difference in the score of CD between those in the control ($\bar{x}$ = 29.00, standard deviation [s.d.] = 0.82) and experimental (CBT) groups ($\bar{x}$ = 28.50, s.d. = 1.29). In addition, participants in the experimental group who were exposed to CBT ($\bar{x}$ = 27.00, s.d. = 0.82) in the second week showed no significant difference in the score of CD compared to those in the control group ($\bar{x}$ = 27.25, s.d. = 1.50). Participants in the experimental group (CBT) reported no significant difference in the 4th and 6th weeks, ($\bar{x}$ = 26.00, s.d. = 0.82) and ($\bar{x}$ = 24.00, s.d. = 1.63), compared to those in the control group ($\bar{x}$ = 27.00, s.d. = 0.82) and ($\bar{x}$ = 26.25, s.d. = 0.96). However, in the 8th week of the intervention, participants in the experimental group (CBT) reported significantly lower scores ($\bar{x}$ = 23.50, s.d. = 0.58) on CD compared to those in the control group ($\bar{x}$ = 26.75, s.d. = 0.50).

**TABLE 1:** Summary of *t*-test showing the significant difference in conduct disorder between the experimental group (cognitive-behavioural therapy) and the control group.

Intervention weeks	Group	*N*	$\bar{x}$	s.d.	*df*	*t*	*p*
Conduct disorder pretest	Control group	4	29.00	0.82	6	0.65	> 0.05
	CBT group	4	28.50	1.29			
Conduct disorder week 2	Control	4	27.25	1.50	6	0.293	> 0.05
	CBT group	4	27.00	0.82			
Conduct disorder week 4	Control	4	27.00	0.82	6	1.732	> 0.05
	CBT group	4	26.00	0.82			
Conduct disorder week 6	Control	4	26.25	0.96	6	2.377	> 0.05
	CBT group	4	24.00	1.63			
Conduct disorder week 8	Control	4	26.75	0.50	6	8.510	< 0.05
	CBT group	4	23.50	0.58			

CBT, cognitive-behavioural therapy; s.d., standard deviation; *df*, degrees of freedom.

The result revealed that CBT had no significant influence on CD amongst in-school adolescents at week 2 (*t*[6] = 0.293, *p* > 0.05), week 4 (*t*[6] = 1.732, *p* > 0.05) and week 6 (*t*[6] = 2.377, *p* > 0.05) of the intervention. There was, however, a significant difference in the level of CD at week 8 of the intervention (*t*[6] = 8.510, *p* < 0.05). This finding supports our first hypothesis. A significant therapeutic effect was observed in week 8 of the intervention.

The finding obtained and summarised in Table 2 showed that at the pretest level, there was no significant difference in the score of CD between those in the control ($\bar{x}$ = 29.00, s.d. = 0.82) and experimental (SST) groups ($\bar{x}$ = 28.50, s.d. = 1.00). In addition, participants in the experimental (SST) group showed no significant difference in the mean ± standard deviation score of CD in the 2nd week ($\bar{x}$ = 26.50, s.d. = 1.29), 4th week ($\bar{x}$ = 26.00, s.d. = 0.82) and 6th week ($\bar{x}$ = 25.26, s.d. = 0.96) compared to those in the control group during the same periods of intervention ($\bar{x}$ = 27.25, s.d. = 1.50); ($\bar{x}$ = 27.00, s.d. = 0.82); ($\bar{x}$ = 25.00, s.d. = 0.82). However, in the 8th week of the intervention, participants in the experimental group (SST) significantly reported lower scores ($\bar{x}$ = 22.25, s.d. = 0.50) on CD compared to those in the control group ($\bar{x}$ = 26.75, s.d. = 0.50). The result revealed SST had no significant influence on CD amongst in-school adolescents at week 2 (*t*[6] = 0.758), *p* > 0.05), week 4 (*t*[6] = 1.732, *p* > 0.05) and at the week 6 of the intervention (*t*[6] = 1.987, *p* > 0.05).

**TABLE 2:** Summary of *t*-test showing the significant difference in conduct disorder between the experimental group (social skills training) and control group.

Intervention weeks	Group	*N*	$\bar{x}$	s.d.	*df*	*t*	*p*
CD pretest	Control group	4	29.00	0.82	6	0.775	> 0.05
	SST group	4	28.50	1.00			
CD week 2	Control	4	27.25	1.50	6	0.758	> 0.05
	SST group	4	26.50	1.29			
CD week 4	Control	4	27.00	0.82	6	1.732	> 0.05
	SST group	4	26.00	0.82			
CD week 6	Control	4	26.25	0.96	6	1.987	> 0.05
	SST group	4	25.00	0.82			
CD week 8	Control	4	26.75	0.50	6	12.728	< 0.05
	SST group	4	22.25	0.50			

CD, conduct disorder; SST, social skills training; s.d., standard deviation; *df*, degrees of freedom.

There was, however, a significant mean ± standard deviation score difference on the level of CD at week 8 of the intervention (*t*[6] = 12.728, *p* < 0.05). This finding supports our second hypothesis, as a significant therapeutic effect was observed in week 8 of the intervention.

The finding obtained and summarised in Table 3 showed that at pretest level, there was no significant difference in the score of CD between those in the control ($\bar{x}$ = 29.00, s.d. = 0.82) and experimental (CBT + SST) groups ($\bar{x}$ = 28.75, s.d. = 0.96). In addition, participants in the experimental (CBT + SST) group showed no significant difference in the mean ± standard deviation score of CD in the 2nd ($\bar{x}$ = 26.75, s.d. = 0.96) and 4th week ($\bar{x}$ = 25.75, s.d. = 1.71), respectively, when compared to those in the control group during same periods of intervention ($\bar{x}$ = 27.25, s.d. = 1.50); ($\bar{x}$ = 27.00, s.d. = 0.82).

**TABLE 3:** Summary of *t*-test showing the significant difference in conduct disorder between the experimental group (cognitive-behavioural therapy and social skills training) and control group.

Intervention weeks	Group	*N*	$\bar{x}$	s.d.	*df*	*T*	*P*
Conduct disorder pretest	Control	4	29.00	0.82	6	0.397	> 0.05
	CBT + SST	4	28.75	0.96			
Conduct disorder week 2	Control	4	27.25	1.50	6	0.562	> 0.05
	CBT + SST	4	26.75	0.96			
Conduct disorder week 4	Control	4	27.00	0.82	6	1.321	> 0.05
	CBT + SST	4	25.75	1.71			
Conduct disorder week 6	Control	4	26.25	0.96	6	2.530	< 0.05
	CBT + SST	4	24.25	1.26			
Conduct disorder week 8	Control	4	26.75	0.50	6	12.728	< 0.05
	CBT + SST	4	22.25	0.50			

CBT, cognitive-behavioural therapy; SST, social skills training; s.d., standard deviation; *df*, degrees of freedom.

However, during week 6 of the intervention, participants in experimental groups (CBT + SST) ($\bar{x}$ = 24.25, s.d. = 1.26) reported significant lower scores on CD compared to those in the control group ($\bar{x}$ = 26.25, s.d. = 0.96). The mean ± s.d. score was more significantly lower on the 8th week of the intervention for participants in experimental group (CBT + SST) ($\bar{x}$ = 22.25, s.d. = 0.50) when compared to those in the control group ($\bar{x}$ = 26.75, s.d. = 0.50).

This result showed that combined CBT and SST showed no significant influence on CD amongst in-school adolescents at week 2 (*t*[6] = 0.562), *p* > 0.05) and week 4 (*t*[6] = 1.321, *p* > 0.05) of the intervention. There was, however, a significant difference in the levels of CD at week 6 (*t*[8] = 2.530, *p* < 0.05) and week 8 (*t*[8] = 12.728, *p* < 0.05) of the intervention. This finding supports our third hypothesis, as significant therapeutic effect was observed in week 6 of the intervention.

The finding obtained in Table 3 showed that at pretest level there was no significant difference in the score of CD between those in the control ($\bar{x}$ = 29.00, s.d. = 0.82) and experimental groups (CBT + SST group) ($\bar{x}$ = 28.75, s.d. = 0.96). In week 2, participants in the experimental group who were exposed to the combined group (CBT + SST) ($\bar{x}$ = 26.75, s.d. = 0.96) reported no significant difference on CD compared to those in the control group ($\bar{x}$ = 27.25, s.d. = 1.50). In addition, in the 4th week, participants in experimental groups (combined group, CBT + SST) ($\bar{x}$ = 25.75, s.d. = 1.71) reported no significant difference on CD compared to those in the control group ($\bar{x}$ = 27.00, s.d. = 0.82).

However, during week 6 of the intervention, participants in experimental groups (CBT + SST) ($\bar{x}$ = 24.25, s.d. = 1.26) significantly reported lower scores on CD compared to those in the control group ($\bar{x}$ = 26.25, s.d. = 0.96). Moreover, in the 8th week of the intervention, participants in experimental groups (CBT + SST) ($\bar{x}$ = 22.25, s.d. = 0.50) significantly exhibited lower scores on CD compared to those in the control group ($\bar{x}$ = 26.75, s.d. = 0.50).

The result confirmed that combined CBT and SST had a significant therapeutic effect on CD amongst in-school adolescents. During week 6 of the intervention, there was a significant difference on the level of CD at week 6 (*t*[8] = 2.530, *p* < 0.05) and week 8 (*t*[8] = 12.728, *p* < 0.05) of the intervention.

The hypothesis that participants in the experimental groups of CBT, SST and CBT and SST combined will exhibit significantly lower levels of CD than the control group is thus fully accepted with the significance of the three packages (CBT, SST and CBT + SST) confirmed.

The result from the Table 4 above showed that there was a significant effect of treatment on groups CBT, SST and CBT + SST, *F*(3, 12) = 10.035, *p* < 0.05, as the experimental groups (CBT, SST and combined CBT and SST) showed a reduction in CD scores across the treatment time (weeks 2, 4, 6 and 8). The main effect comparing the time of treatment (weeks 2, 4, 6 and 8) was significant, *F*(4, 48) = 56.697, *p* ≤ 0.05, indicating that there was a significant difference between the scores of participants who are in the treatment groups and participants in control group on CD across the periods of intervention (weeks 2, 4, 6 and 8). There was a significant interaction between treatment and therapeutic time, *F*(12, 48) = 2.733, *p* < 0.05.

**TABLE 4:** Summary of 2-way analysis of variance for repeated measures showing the difference in the most efficacious psychotherapies (cognitive-behavioural therapy, social skills training and combined cognitive-behavioural therapy + social skills training) in the treatment of conduct disorder amongst in-school adolescents.

Source	Sum of squares	*df*	Mean square	*F*	Sig.
**Between-subjects**					
Group	33.837	3	12.746	10.035	< 0.05
Error	15.250	12	1.271		
**Within-subjects**					
Time	38.237	4	58.469	56.697	< 0.05
Time * Treatment	33.825	12	2.819	2.733	< 0.05
Error (time)	49.500	48	0.031		

CBT, cognitive-behavioural therapy; SST, social skills training; *df*, degrees of freedom.

As summarised in Table 5, our research findings show that the three therapeutic techniques, CBT, SST, and CBT + SST, are independently effective in managing CD amongst in-school adolescents. However, CBT + SST appear to be more significantly effective in the management of CD.

**TABLE 5:** Showing the *post hoc* analysis on the efficacy of cognitive-behavioural therapy, social skills training and combined cognitive-behavioural therapy and social skills training in the treatment of conduct disorder amongst in-school adolescents.

(I) Groups	(J) Groups	Mean difference (I-J)	Std. Error	Sig.
Control group	CBT	1.45*	0.36	0.002
	SST	1.60*	0.36	0.001
	CBT + SST	1.70*	0.36	0.000
CBT	Control group	−1.45*	0.36	0.002
	SST	0.15	0.36	0.681
	CBT + SST	0.25	0.36	0.496
SST	Control group	−1.60*	0.36	0.001
	CBT	−0.15	0.36	0.681
	CBT + SST	0.10	0.36	0.784
CBT + SST	Control group	−1.70*	0.36	0.000
	CBT	−0.25	0.36	0.496
	SST	−0.10	0.36	784

CBT, cognitive-behavioural therapy; SST, social skills training.

* , significant at 0.01 level.

Findings from Table 5 show that the three therapeutic techniques CBT, SST and CBT and SST combined are independently effective in the management of CD amongst in-school adolescents. However, CBT + SST shows itself to be significantly more effective in the management of CD amongst in-school adolescents in Ibadan. This thus confirmed the fourth hypothesis, which states that combined CBT and SST will be more efficacious than each treatment received alone.

## Discussion

The result of the first hypothesis shows that there was a significant difference in the level of reduction of CD amongst participants in the experimental (CBT) group and those in the control group. This result corroborates the findings of Wolinsky and Miller,^22^ who found that cognitive training would affect the cognitive ability targeted by that training, and these effects would be maintained over time. It also indicates that maintained improvements in cognitive ability would have a positive transfer effect on everyday function. Conduct problems tend to be particularly treatment-resistant,^23^ underscoring the need for high-quality interventions with documented outcome effects when treating this population. This study finding also corroborates the findings of Gardner,^24^ who confirmed the effectiveness of CBT in treating rebelliousness, delinquency and CD. According to Gardner,^24^ cognitive factors play an essential role in involvement with undesirable behaviours. Thus, replacing negative habits with positive ones and rethinking them will equally help individuals generate more adaptive behaviour.

**FIGURE 1:**
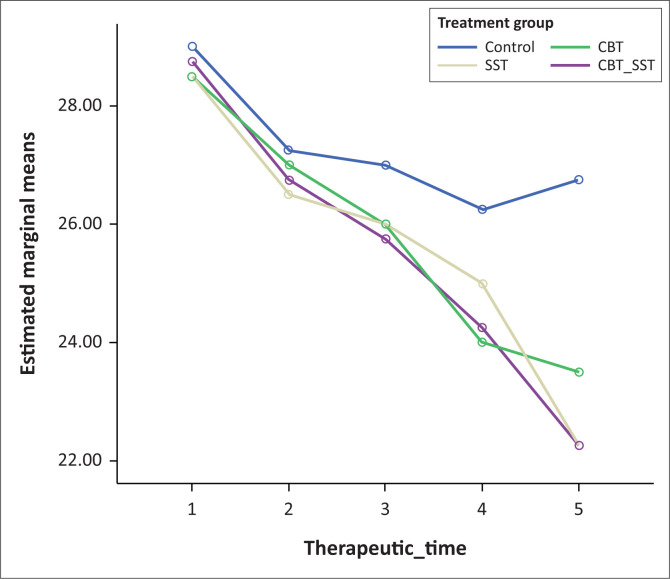
The efficacy of the behavioural interventions (CBT, SST and CBT+SST) on conduct disorder.

The result of the second hypothesis showed that there was a significant difference in the level of reduction of CD of participants in the experimental (SST) and those in the control group. This affirms the findings of National Institute for Health and Care Excellence (NICE)^25^ that programmes like SST are effective individualised therapy. In a recent meta-analysis study, SST has been shown to improve the problems of adolescent behaviour and the social functioning and family interaction of the adolescent.^26^ Social skills trainings have been included in some meta-analyses that examined the effectiveness of offender treatment.^17,27,28,29^ Some other research studies have been carried out on SST for emotionally and behaviourally disturbed juveniles,^13,30,31^ with generally positive overall treatment effects. When taught skills were assessed, it was found that training for social skills showed moderate efficacy in reducing antisocial behaviour.^32^ Still, it has been noted that there is a need for long-term efficacy studies.^33^ This finding explains why it took eight weeks of intervention before a significant difference in levels of CD was observed.

The result of the third hypothesis showed that there was a significant difference in the level of CD amongst participants in the experimental CBT + SST group compared to those in the control group. Although SST has demonstrated efficacy for individuals with conduct problems when used in isolation, research indicates that an individual who has conduct problems is likely to show more significant improvements when using SST in conjunction with other treatment methods.^34^ Research has also further shown that using SST and CBT concurrently when treating individuals with CD enhances adaptive changes.^35,36,37^ The result of this study further verifies both interventions were more effective together in the treatment of CD.

The result of this hypothesis is an affirmation of the theory and previous studies that were carried out on CBT and SST.^8,38,39^ With the aid of CBT, clients are assisted in reconsidering any maladaptive pattern in their thinking-feeling-behaviour cycles. A client’s goal is to rethink these patterns and reconsider more adaptive alternatives that would work better for them. These skills involved in the above process are what the adolescents in the experimental I (CBT) group have been exposed to. The adolescents in the experimental II (SST) group were also exposed to the nitty-gritty of SST, aiming to provide a method for structuring and orchestrating modelling opportunities. The privilege of role-playing and reversing roles is to help adolescents better understand their present behaviours and consequently enhance the desire for a positive change. The foregoing also supports the fourth hypothesis, which states that combined CBT and SST will be more efficacious than each treatment received alone.

As an implication for further study, it was revealed that combined CBT and SST is more effective in terms of therapeutic time, as there was a reduction in CD after 6 weeks of psychotherapeutic intervention. Independently, CBT and SST participants reported a reduction after 8 weeks in treatment. This implies CD requires a more prolonged therapeutic time for a change of behaviour to occur.

## Conclusion and recommendation

This study investigated the efficacy of CBT and SST on CD amongst in-school adolescents in Ibadan. It was observed that combined psychotherapeutic treatment (CBT + SST) was more effective than CBT or SST independently. Because CBT and SST have been tested and found effective in treating CD in adolescents, it is recommended that the use of these two psychobehavioural interventions be encouraged to combat CD. A larger sample size is also recommended to further establish the findings of this research.

## Limitation of study

The study should have lasted for another three months, which could not take place because of some students changing their school and graduating from secondary school. A follow-up would have helped determine if there was a total elimination of CD. Also, it is a rule of thumb that an experiment should have a minimum sample size of 30; however, the constraints of getting more participants to stay through the sessions for the intervention limited the sample size in this research to 16. This also can be seen as a limitation to this study.
